# Adherence to standards of first-visit antenatal care among providers: A stratified analysis of Tanzanian facility-based survey for improving quality of antenatal care

**DOI:** 10.1371/journal.pone.0216520

**Published:** 2019-05-13

**Authors:** Deogratius Bintabara, Keiko Nakamura, Julius Ntwenya, Kaoruko Seino, Bonaventura C. T. Mpondo

**Affiliations:** 1 Department of Global Health Entrepreneurship, Division of Public Health, Graduate School of Tokyo Medical and Dental University, Tokyo, Japan; 2 Department of Public Health, College of Health Sciences, The University of Dodoma, Dodoma, Tanzania; 3 Department of Environmental Health Sciences, School of Public Health, University of Michigan, Ann Arbor, Michigan United States of America; 4 Department of Internal Medicine, College of Health Sciences, The University of Dodoma, Dodoma, Tanzania; Hamamatsu Ika Daigaku, JAPAN

## Abstract

**Introduction:**

Despite the benefits of early antenatal care visits for early prevention, detection, and treatment of potential complications in pregnancy, a high level of provider adherence to first-visit antenatal care standards is needed. However, little information is available regarding provider adherence to antenatal care in Tanzania. This study was performed to assess provider adherence to first-visit antenatal care standards and to apply stratified analysis to identify associated factors in Tanzania.

**Methods:**

Data from the 2014–2015 Tanzania Service Provision Assessment Survey were used in this study. Provider adherence to first-visit antenatal care standards was measured using 10 domains: client history; aspects of prior pregnancies; danger signs of the current pregnancy; physical examination; routine tests; HIV testing and counseling; maintaining a healthy pregnancy; iron/folate supplements; tetanus toxoid vaccination, and preparation for delivery. A composite score was then created in which the highest quantile (corresponding to ≥60.5%) considered to provider adhering to first-visit antenatal care standards. Initially, a series of unadjusted logistic regression analyses according to the type of facility and managing authority were performed separately at each level (i.e., facility, provider, and client). Thereafter, all variables with *P* < 0.2 were fitted into the respective stratified multivariable logistic regression analysis using a 5% significance level.

**Results:**

A total of 1756 first-visit antenatal care consultations performed by 822 providers in 648 health facilities were analyzed. The overall median [Interquartile range, IQR] adherence to first-visit antenatal care was relatively low at 47.1% [35.7%–60.5%]. After adjusting for selected variables from each level in specific strata, at dispensary; female providers [AOR = 5.5; 95% CI, 1.8–16.4], at health centre; performance of quality assurance [AOR = 2.2; 95% CI, 1.3–3.9], at hospital; availability of routine tests [AOR = 2.5; 95% CI, 1.3–4.8] and basic medicine [AOR = 2.8; 95% CI, 1.4–5.7], at public facilities; availability of medicine [AOR = 1.8; 95% CI, 1.1–3.2] and receiving refresher training [AOR = 1.8; 95% CI, 1.1–3.1], and at private facility; receiving external fund from government [AOR = 3.0; 95% CI, 1.1–8.4] were significantly associated with better adherence to first-visit antenatal care standards.

**Conclusions:**

The study highlighted the important factors, including the provision of refresher training, regular distribution of basic medicines, and diagnostics equipment which may influence provider adherence to first-visit ANC standards.

## Introduction

Over the past 25 years, the maternal mortality ratio (MMR) has been decreasing in almost all Millennium Development Goal (MDG) regions [1]. However, low and middle-income countries (LMIC) still account for nearly 99% of the global MMR, with Sub-Saharan Africa (SSA) contributing approximately 66% of all such mortalities [1,2]. In Tanzania, the reported MMR is still high (556 maternal deaths per 100 000 live births) and the decreasing trend has remained unchanged over the past decade [3,4]. This high MMR is far from the operational targets set by Tanzania’s Ministry of Health, Community Development, Gender, Elderly and Children (MoHCDGEC) through the National Road Map Strategic Plan of 2008, which aimed to accelerate reduction to 193 maternal deaths per 100 000 live births by the end of 2015 [5]. This high MMR has raised concerns regarding whether Tanzania will achieve Sustainable Development Goal (SDG) 3 by the end of 2030, which seeks to ensure healthy lives and promote wellbeing for people of all ages, while addressing major health priorities, including maternal and child health [6].

Several reports have suggested that antenatal care (ANC) has no or only a minimal direct effect on reducing MMR [7–9]. However, there is new evidence that ANC does play an important role in minimizing maternal and neonatal mortality [10,11]. Therefore, the World Health Organization (WHO) has promoted a focused ANC model that was developed in the 1990s [12,13] as a strategy to reduce the burden of maternal and perinatal mortality, especially in LMIC [14,15]. This model places a great deal of emphasis on the quality of clinical services, with the recommendation that all pregnant women should attend a minimum of four ANC visits and receive structured preventive interventions during each visit [16–18]. However, secondary analysis of previously collected data indicated that the model including four ANC visits is associated with higher rates of perinatal death than ANC model requiring at least eight visits [19]. Based on these and other findings, the WHO developed the new 2016 ANC model, which recommends a minimum of eight ANC visits consisting of one visit in the first trimester, two visits in the second trimester, and five visits in the third trimester [20].

Despite these new WHO ANC recommendations, the MoHCDGEC policy in Tanzania is still implementing the previous model requiring four focused ANC visits [21,22]. Implementation of the focused ANC model since 2002 in Tanzania has helped to increase ANC coverage, which has exceeded 90% for at least two decades in this country. In 2016, about 98% of women age 15–49 years received ANC from a skilled provider during the pregnancy of their most recent birth [3,4]. However, despite the explained benefits of early and frequent ANC visits to increase the likelihood of early prevention, detection, and treatment of potential pregnancy complications [23], the proportions of pregnant women beginning ANC in their first trimester (24%), those who complete four or more ANC visits (51%), and births assisted by skilled providers (64%) are still unsatisfactory [4]. Improving the quality of the first ANC visit, thus reaching almost all pregnant women in Tanzania, will help to increase their level of satisfaction, and therefore provide motivation to attend follow-up visits.

Although a complex issue, several measures are available for assessing the quality of health care that can be divided into three broad categories: structure attribute, which can be used to assess the characteristics of care settings; process attribute, involving assessment of the services provided to patients; and outcome attribute, that evaluates patient health resulting from the care provided [24,25]. There have been a number of reports involving the assessment of ANC quality using one or all of these measures [26–28]. Furthermore, there is the minimal usage of the process attribute measure, which includes direct observation of items or consultations and has been mentioned as the gold standard, to assess the quality of care [29]. Although this attribute can be used to determine whether the services provided to patients are consistent with the routine standard of clinical care until recently assessment of ANC quality has mostly focused on quantifiable issues, such as the number of visits and the timing of the first visit [30]. There is, therefore, a paucity of data regarding the quality of health services received by pregnant women at a single point of contact with health providers, especially with regard to the first ANC visit, as it tends to cover almost all pregnant women in many low-resource countries, such as Tanzania.

There have been a number of studies to assess the quality of ANC in Tanzania based on timing, the number of ANC visits, availability of basic equipment, or client satisfaction [31–33]. However, only a few studies assessed the quality of ANC based on the process attribute measure [34–36]. But these studies did not assess factors associated with the quality of services offered by ANC providers. Furthermore, limited empirical evidence is available regarding the extent to which providers adhere to the first-visit ANC standards and associated factors in Tanzania. Therefore, the present study was performed to assess the adherence to first-visit ANC standards among providers and to perform stratified analysis to identify factors associated with service provider adherence to first-visit ANC standards across different strata of health facilities (dispensary/clinic, health center, hospital) and health facility managing authority (public and private). The findings of the present study will help to improve provider adherence to the first-visit ANC and to ensure that all pregnant women receive good quality care.

## Materials and methods

### Data source

Data from the 2014–2015 Tanzania Service Provision Assessment (TSPA) Survey were used in the present study. The TSPA Survey was performed by Tanzania’s National Bureau of Statistics (NBS) in collaboration with the Office of the Chief Government Statistician (OCGS), Zanzibar, the MoHCDGEC, Tanzania Mainland, and the Ministry of Health (MOH), Zanzibar. Technical support for the survey was provided by ICF International under the Demographic and Health Survey (DHS) program. The survey was designed to provide information on the quality, service readiness and availability of basic and essential health care services. The survey also assessed the presence and functioning of components essential for quality service delivery for child health, maternal and newborn care, family planning, antenatal care, sexually transmitted infections, HIV/AIDS and non-communicable diseases.

### Study sample and sampling procedure

**Fig 1:**
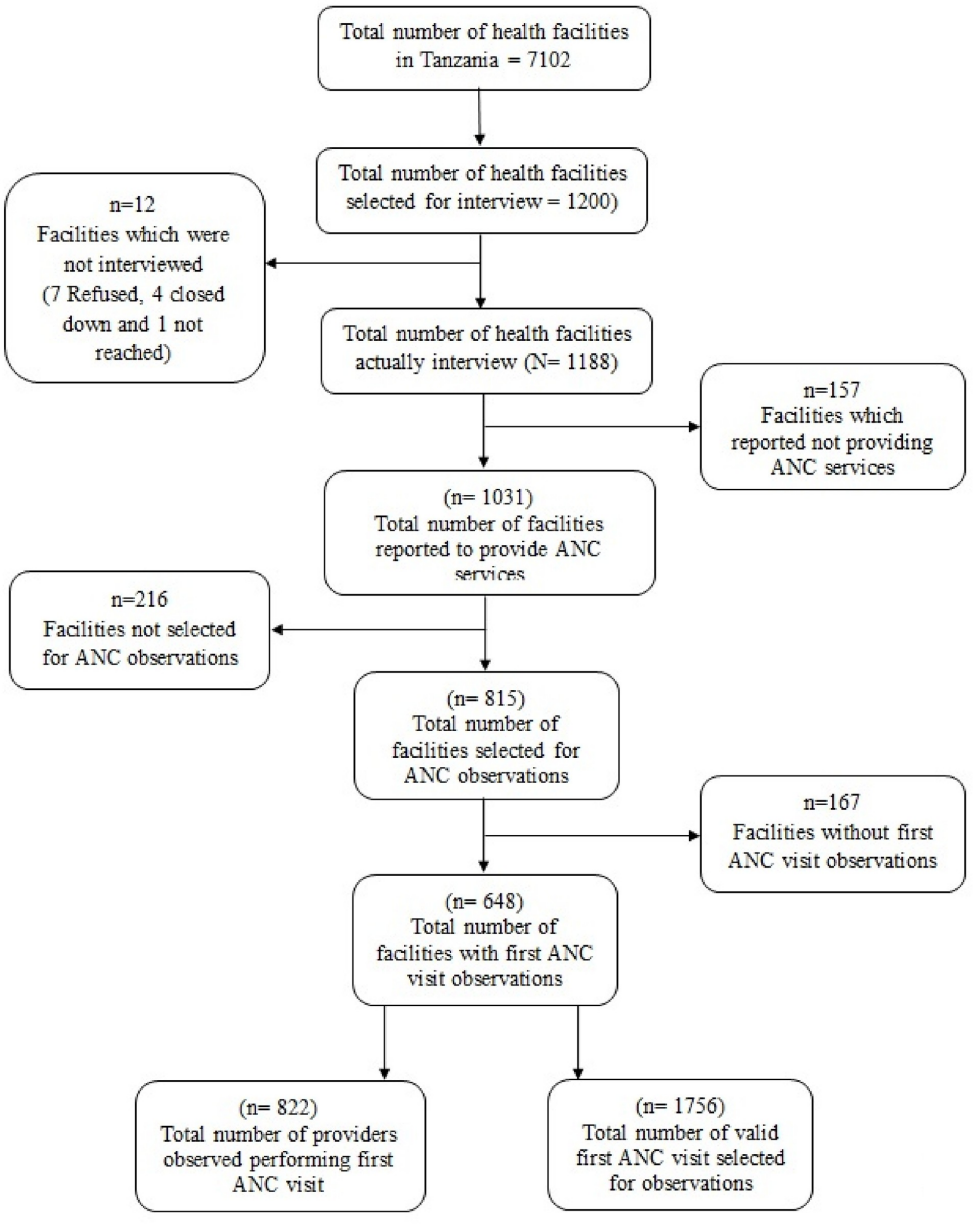
Selection procedure for the sampling units included in this analysis.

The 2014–2015 TSPA was a sample survey of all formal sector health facilities in Tanzania. The MoHCDGEC in Tanzania mainland and MOH in Zanzibar provided lists of all hospitals, health centers, dispensaries, and clinics, which were compiled into a national master facility list (sampling frame). A nationally representative sample of 1200 facilities was selected from this national master facility list by random stratified sampling according to health facility type, managing authority, and region. Further details about the TSPA survey sampling procedures are available online [37]. However, for the purpose of our research, the analysis was restricted to health facilities with at least one first ANC visit consultation. Therefore, facilities that reported providing ANC services, selected to participate for ANC observation, were open on the day of the interview, and agreed to participate were eligible and were included in this study. Facilities that did not fulfill the inclusion criteria were excluded from the study: 157 did not provide ANC services, 216 were not selected for ANC observation, 167 did not have first ANC visit observations, seven refused to participate, four were closed on the day of the interview, and one could not be reached. After excluding all facilities that did not fulfill the inclusion criteria, 648 health facilities that corresponded to 1756 first ANC visits performed by 822 health providers were included in this study (Fig 1).

In each selected facility, clients were identified and systematically selected for observation based on the number of clients present at each service site on the day of the visit. In cases where many clients were present and eligible for observation, a maximum of five clients for each provider and a maximum of 15 observations for each selected facility were selected by systematic random sampling [36].

### Data collection and processing

The 2014–2015 TSPA used four main data collection tools: a Facility Inventory; a Health Provider Interview questionnaire; Observation Protocols and Exit Interview questionnaire. The data for the original survey was collected between October 20, 2014, and February 21, 2015, and some facilities that were not interviewed previously were revisited between March 2 and 13, 2015. Data collection were performed by health workers who were selected after a series of training, practicals, and examinations to qualify as interviewers. The information collected from each questionnaire was stored in different files except Observation Protocols and Exit Interview that were stored in the same file. The data in these three files were edited, cleaned, and recoding was performed for variables of interest. The files were then linked into a new dataset using the facility and provider identification as a unique identifiers. The survey final report, which is available online, provides more descriptions of the TSPA survey design, sampling procedures, and data collection [36].

### Definitions of variables

#### Outcome variable

Provider adherence to first-visit ANC standards was determined based on the responses documented by an observer/interviewer regarding whether each of the required services was performed during the first ANC visit. Interviewers were required to note whether providers discussed or performed 53 items grouped into 10 domains related to first-visit ANC services as proposed by MoHCDGEC and other surveys in Tanzania [21,36,38]. These domains, which were determined by the survey designers, included: client history (four items); aspects of prior pregnancies (10 items); danger signs of the current pregnancy (seven items); physical examination (eight items); routine tests (four items); HIV testing and counseling (five items); maintaining a healthy pregnancy (three items); iron/folate (FeFo) supplements (four items); tetanus toxoid vaccination (three items); and preparation for delivery (five items). For each directly observed visit, the responses for all items in these ten domains were aggregated to calculate the adherence score as the percentage of actions completed among the total items assessed [39]. Each domain accounted for 10% as the target was 100% (i.e., 100%/10). The percentage of each item within the domain was equal to 10% divided by the number of items in that domain. A similar approach involving assigning an equal weight for each domain and the indicators within it has been used in other studies in different fields [40,41]. The ANC adherence score for each observation was then calculated by summing the percentages. This approach has also been used elsewhere [40]. Based on previous reports, all observations were classified into the highest quantile (corresponding to ≥ 60.5%) of the score as providers that adhered to ANC standards [42]. The items in each domain are listed and the measurement process is summarized in more details in S1 Table.

#### Independent variables

The independent facility variables were facility location, managing authority, facility type, quality assurance (i.e., any quality assurance activities carried out during the past year), external sources of funding, basic equipment, routine tests, basic medicines, and ANC guidelines. The independent provider variables were sex, cadre, working experience, ANC refresher training, and supportive supervision. Independent client variables were age and level of formal education. These variables are summarized in S2 Table.

### Statistical analysis

**Fig 2:**
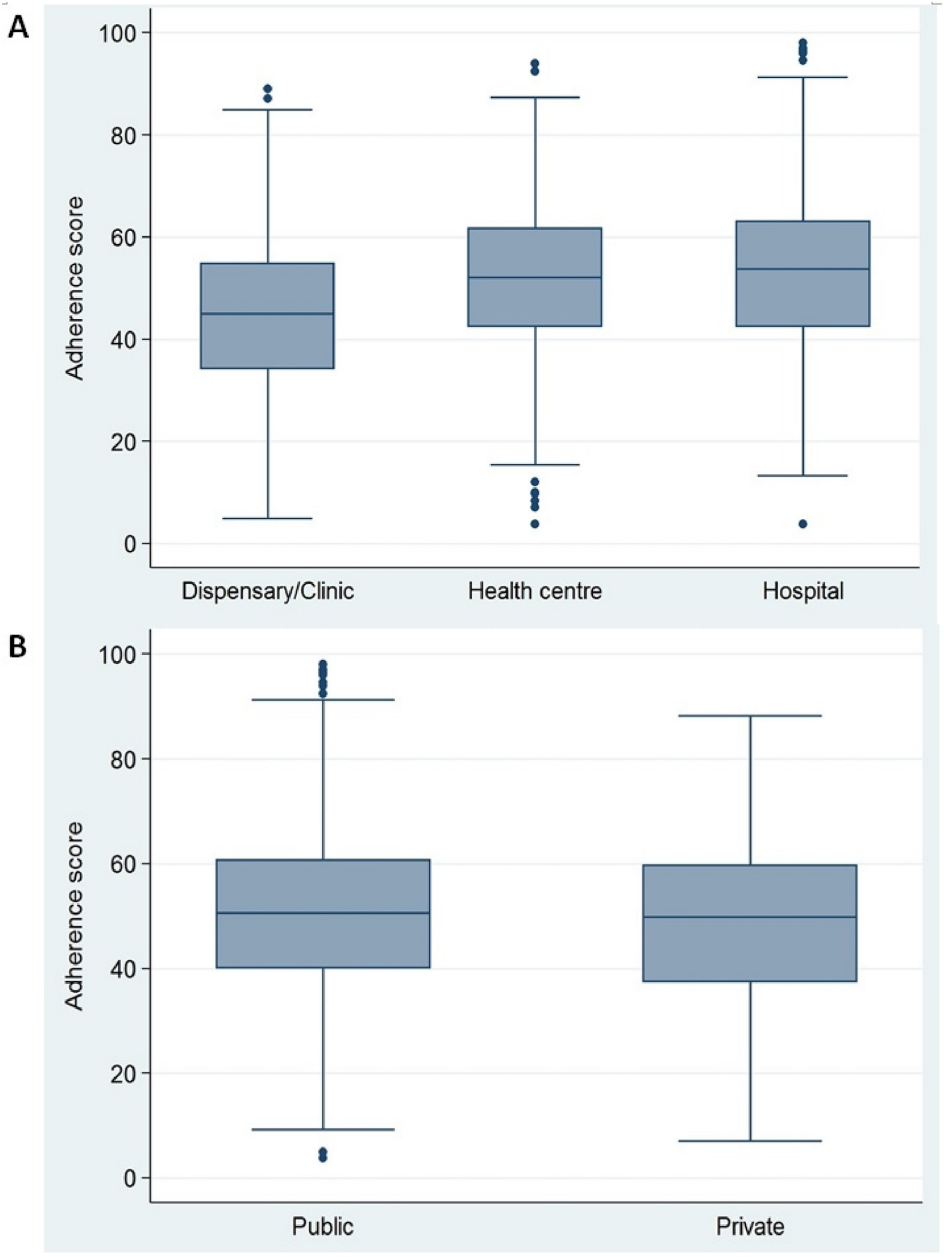
Distribution of adherence scores for observed first-visits ANC in TSPA 2015–2016 (*n* = 1756). (A) Provider adherence to first-visit ANC standards according to facility type. (B) Provider adherence to first-visit ANC standards according to managing authority. Boxes show the limits of 25th and 75th percentiles. Horizontal lines inside the boxes show the median values. Bars show the lower and upper limits of 95% CI. The dots show outliers.

In descriptive analyses, categorical variables were summarized using weighted frequencies and proportions (Tables 1 and 2, and S2 Table), while continuous variables were summarized using the median and interquartile range (IQR) (Table 3 and Fig 2). Stratified descriptive analyses were performed to assess the differences in availability of ANC services according to facility type and managing authority. Furthermore, a series of individual unadjusted logistics regression analyses were constructed across different strata of health facilities (dispensary/clinic, health center, hospital, public) and health facility managing authority (public or private) to identify factors separately at each level—facility (1a, 2a, 3a, 4a, and 5a), provider (1b, 2b, 3b, 4b, and 5b), and client (1c, 2c, 3c, 4c, and 5c)—that were associated with provider adherence to first-visit ANC standards. Thereafter, all independent variables that showed an association with P < 0.2 at any of defined strata of facility type and/or managing authority were eligible for inclusion in multivariable logistics regression analyses 1d, 2d, 3d, 4d, and 5d, respectively, adjusting for all three level factors. All models were fitted using a stepwise (backward) elimination method and P < 0.05 was taken to indicate statistical significance. The odds ratio (OR) and 95% confidence interval (95% CI) for each variable were computed and used to measure the association with the outcome variable. As the original TSPA survey used a random stratified sampling method, this approach may over- or underestimate the sample within the strata. Therefore, all analyses took this complex survey design into account. All statistical analyses were performed using Stata 14 (StataCorp, College Station, TX).

**Table 1:** Baseline characteristics, facility, provider, and client distributions in TSPA 2014–15.

Variable	n (%)
**Facility characteristics (n = 648)**	
**Facility location** (Rural)	510 (78.7)
**Facility type**	
Clinic & dispensary	519 (80.1)
Health centre	94 (14.5)
Hospital	35 (5.4)
**Managing authority** (Public)	535 (82.6)
**Quality assurance** (Performed < 1 year)	126 (19.4)
**External source of funding** (Government)	431 (66.5)
**Provider characteristics (n = 822)**	
**Provider’s sex** (Female)	697 (84.8)
**Cadre** (Nurse)	776 (94.4)
**Refresher training** on ANC (Received)	367 (44.6)
**Supportive supervision**	
Not received	148 (18.0)
Received < 3 months	555 (67.5)
Received > 3 months	119 (14.5)
**Working experience**	
< 2 years	327 (39.8)
2–5 years	185 (22.5)
> 5 years	310 (37.7)
**Client characteristics (n = 1756)**	
**Age**	
< 20 years	332 (18.9)
20–35 years	1235 (70.3)
> 35 years	189 (10.8)
**Education level**	
None	409 (23.3)
Primary	1084 (61.7)
secondary and above	263 (15.0)

**Table 2:** Percentage distribution of ANC service availability according to facility type and managing authority in TSPA 2014–2015 (*n* = 648).

Variable	Type of facility			Managing authority		Total n (%)
	Dispensary n (%)	Health centre n (%)	Hospital n (%)	Public n (%)	Private n (%)	
**Staff and training**						
Presence of guidelines ^t^	278 (53.6)	68 (72.3)	26 (74.3)	308 (57.6)	64 (56.6)	372 (57.4)
Availability of trained staff ^t^	327 (63.0)	75 (79.8)	31 (88.6)	352 (65.8)	81 (71.7)	433 (66.8)
**Equipment**						
Blood pressure apparatus ^t m^	399 (76.9)	81 (86.2)	33 (94.3)	414 (77.4)	99 (87.6)	513 (79.2)
**Diagnostics**						
Hemoglobin test	81 (15.6)	26 (27.7)	9 (25.7)	95 (17.8)	21 (18.6)	116 (17.9)
Urine for protein test	71 (13.7)	19 (20.2)	8 (22.9)	76 (14.2)	22 (19.5)	98 (15.1)
**Medicines and commodities**						
Iron tablets	466 (89.8)	83 (88.3)	33 (94.3)	489 (91.4)	93 (82.3)	582 (89.8)
Folic acid tablets ^m^	481 (92.7)	84 (89.4)	32 (91.4)	503 (94.0)	94 (83.2)	597 (92.1)
Sulfadoxine+Pyrimethamine (SP)	267 (51.4)	49 (52.1)	21 (60.0)	286 (53.5)	51 (45.1)	337 (52.1)
Tetanus toxoid vaccine ^t^	446 (85.9)	86 (91.5)	34 (97.1)	466 (87.1)	100 (88.5)	566 (87.3)
Insecticide-treated bed nets	30 (5.8)	12 (12.8)	3 (8.6)	37 (6.9)	8 (7.1)	45 (6.9)
**Total**	519	94	35	535	113	648

Note:

^*t*^ = *P* < 0.05 according to the type of facility.

^*m*^ = *P* < 0.05 according to managing authority.

^*t m*^ = *P* < 0.05 according to both type of facility and managing authority.

The *P*-values presented here were obtained with the Chi-square test.

The items presented here were assessed in the ANC service area.

**Table 3:** Overall and domain-specific scores of provider adherence to first-visit ANC standards in TSPA 2014–2015.

Variable	Median (IQR)% All pregnant women (n1756)	Median (IQR)% Women ≥ 2 pregnancies (n = 1301)
Client history	75.0 (50.0–75.0)	75.0 (50.0–75.0)
Aspects of prior pregnancies	10.0 (0.0–30.0)	20.0 (10.0–40.0)
Danger signs of the current pregnancy	42.9 (0.0–57.1)	42.9 (0.0–57.1)
Physical examination	50.0 (37.5–75.0)	50.0 (37.5–75.0)
Routine tests	25.0 (0.0–100.0)	25.0 (0.0–100.0)
HIV Testing and Counseling	80.0 (40.0–100.0)	80.0 (40.0–100.0)
Maintain health pregnancy	33.3 (0.0–66.7)	33.3 (0.0–66.7)
Iron/ Folate (FeFo) supplementation	75.0 (50.0–75.0)	75.0 (50.0–75.0)
Tetanus toxoid injection	33.3 (33.3–66.7)	33.3 (33.3–66.7)
Preparation for delivery	40.0 (20.0–80.0)	40.0 (0.0–80.0)
Adherence score quantile		
Low	31.0 (24.8–35.3)	31.2 (25.6–35.9)
Second	45.7 (42.8–47.9)	45.7 (43.1–47.9)
Third	54.6 (52.5–57.5)	54.7 (52.7–57.5)
High	68.0 (63.7–74.2)	68.2 (63.7–74.4)
Overall score	47.1 (35.7–60.5)	47.8 (36.3–60.7)

### Ethical considerations

This study was based on analysis of existing public domain survey (TSPA 2014–2015) datasets that are freely available online with all identifying information removed. The original survey was approved by the Ethics Committee of the ICF Macro at Calverton in the USA and by the National Institute of Medical Research Ethics Committee in Tanzania. Therefore, ethical approval for this analysis was deemed unnecessary. Informed consent was requested and obtained from participants before the interview.

## Results

### Background characteristics of facilities, providers, and clients

Table 1 summarized the background characteristics of the health facilities, health providers, and clients included in the survey. More than two-thirds of the 648 health facilities were located in rural settings, and more than 80% were publicly owned facilities. About 95% of all 822 health providers were nurses, while less than half had received ANC refresher training within the previous 24 months. Nearly three-quarters of 1756 observed first-visit ANC consultations involved women between the ages of 20 and 35 years. For stratified descriptive statistics, see S2 Table.

### Availability of ANC services

Table 2 shows the distribution of ANC service availability according to facility type and managing authority. The availability of ANC guidelines and at least one staff member who received refresher training were significantly low at the dispensary/clinic level (P < 0.05). However, the availability of diagnostic tools (Hb and urine tests) was low across all facility and managing authority types. The overall availability of basic medicines was high, with folic acid tablets being more prominent at private facilities and tetanus toxoid vaccine more prevalent at hospitals (P < 0.05).

### Provider adherence to first-visit ANC standards

Table 3 shows the median (IQR) of the 10 predetermined domains used to assess overall adherence to first-visit ANC standards among health providers. Although the overall median adherence score was relatively low (47.1%), providers had high adherence in some separate individual domains, such as client history (75.0%), discussion or provision of FeFo supplements (75.0%), and HIV testing and counseling (80.0%). When the adherence score was disaggregated according to facility type and managing authority, the overall median adherence score was low for dispensaries/clinics (Fig 2A), while there was no significant difference between public and private facilities (Fig 2B).

### Factors associated with provider adherence to first-visit ANC standards

Table 4 shows the results of a series of stratified logistics regression models to examine the associations between selected independent variables and adherence with first-visit ANC standards. At the dispensary level (model 1a–c) and the corresponding model 1d that adjusted for all selected factors from all three facility levels, the odds of adherence to first-visit ANC standards were five and three times higher among females and clinician providers compared to males and nurses, respectively. At the health centre level (model 2a–c) and the corresponding model 2d that adjusted for all selected factors from all three facility levels, the odds of adherence to ANC standards were two times higher among providers in the facilities that regularly performed quality assurance. At the hospital level, model 3a–c and the corresponding model 3d that adjusted for all selected factors, the odds of adherence to ANC standards were two and three times higher for providers in the facilities that performed routine tests and had basic medicines, respectively, and also five times higher for providers with less than 2 years of working experience compared to those with more than 2 years of experience. In publicly owned facilities, model 4a–c and the corresponding model 4d that adjusted for all selected factors, the odds of adherence to first-visit ANC standards were four times higher among female providers and two times higher among providers who had received refresher training and those in facilities that had basic medicines. In privately owned facilities, model 5a–c and the corresponding model 4d that adjusted for all selected factors, the odds of adherence to ANC standards were three times higher among privately owned facilities that received some funding from the government compared to those had not received such funding. Furthermore, in all strata except at the health center level, the odds of adherence to ANC standards were higher when providers provided services for women between 20 and 35 years old.

**Table 4:** Stratified logistics regression models for factors associated with adherence to first-visit ANC standards in TSPA 2014–2015 (*n* = 1756).

Variable	Facility type						Managing authority			
	Dispensary/Clinic		Health center		Hospital		Public		Private	
	OR [95%CI]		OR [95%CI]		OR [95%CI]		OR [95%CI]		OR [95%CI]	
	**Model 1a**	**Model 1d**	**Model 2a**	**Model 2d**	**Model 3a**	**Model 3d**	**Model 4a**	**Model 4d**	**Model 5a**	**Model 5d**
**Facility factors**										
**Facility location**										
Urban (versus rural)	1.6 [0.6–3.9]		0.9 [0.5–2.0]		1.2 [0.6–2.3]		1.4 [0.8–2.5]		1.3 [0.6–2.9]	
**Quality assurance**										
Performed (versus not performed)	2.0 [0.7–5.8]	1.7 [0.6–5.4]	2.3 [1.3–4.1]	**2.2 [1.3–3.9]**	0.3 [0.1–0.8]	0.3 [0.2–1.0]	1.9 [1.0–3.7]	1.8 [1.0–3.3]	1.2 [0.4–3.6]	
**ANC guideline**										
Available (versus not available)	0.7 [0.3–1.8]		1.0 [0.4–2.0]		1.5 [0.7–3.3]		1.0 [0.5–2.0]		0.5 [0.2–1.2]	0.5 [0.2–1.2]
**Basic equipment**										
Available (versus not available)	1.2 [0.4–3.2]		0.9 [0.4–2.0]		1.7 [0.7–4.4]		1.1 [0.5–2.5]		1.6 [0.4–6.8]	
**Routine tests**										
Available (versus not available)	0.4 [0.1–4.4]		1.3 [0.6–2.8]		2.3 [1.2–4.5]	**2.5 [1.3–4.8]**	1.2 [0.6–2.7]		1.0 [0.4–2.4]	
**Basic medicines**										
Available (versus not available)	2.2 [1.1–4.8]	2.2 [1.0–4.8]	1.2 [0.7–2.2]		2.6 [1.3–5.3]	**2.8 [1.4–5.7]**	1.8 [1.1–3.2]	**1.8 [1.1–3.2]**	2.4 [1.0–5.4]	
**External fund**										
Government (versus non-government)	1.3 [0.6–2.9]		0.7 [0.4–1.4]		3.5 [1.1–11.4]	2.4 [0.9–6.4]	1.0 [0.6–1.9]		3.0 [0.9–9.8]	**3.0 [1.1–8.4]**
**Provider factors**	**Model 1b**		**Model 2b**		**Model 3b**		**Model 4b**		**Model 5b**	
**Provider’s sex**										
Female (versus male)	6.0 [2.1–17.5]	**5.5 [1.8–16.4]**	1.7 [0.6–4.8]		0.9 [0.3–3.1]		4.4 [1.9–10.5]	**3.8 [1.6–9.0]**	1.5 [0.3–8.4]	
**Cadre**										
Nurse (versus clinician)	0.3 [0.1–0.9]	**0.3 [0.1–0.9]**	1.3 [0.2–7.4]		15.0 [1.8–126.9]	7.9 [0.9–71.3]	0.4 [0.2–1.1]	0.4 [0.1–1.0]		
**Refresher training**										
Received (versus not received)	1.7 [0.9–3.5]	1.9 [0.9–4.0]	1.7 [0.9–3.1]	1.4 [0.8–2.4]	1.6 [0.8–3.1]	1.7 [0.8–3.6]	1.7 [1.0–2.9]	**1.8 [1.1–3.1]**	1.2 [0.5–2.7]	
**Supportive supervision**										
Not received	1.0		1.0		1.0		1.0		1.0	
Received < 3 months	1.6 [0.5–4.9]		1.0 []0.5–2.1		0.9 [0.4–1.9]		1.1 [0.6–2.2]		1.3 [0.4–4.5]	
Received > 3 months	1.2 [0.3–4.3]		1.1 [0.4–2.9]		0.9 [0.4–2.1]		1.0 [0.4–2.3]		0.8 [0.2–3.1]	
**Working experience**										
< 2 years	1.0	1.0	1.0		1.0	1.0	1.0	1.0	1.0	
2–5 years	0.5 [0.2–1.3]	0.5 [0.2–1.3]	0.8 [0.3–1.9]		0.2 [0.1–0.7]	**0.2 [0.1–0.6]**	0.5 [0.2–1.0]	0.5 [0.2–1.0]	0.8 [0.2–3.3]	
> 5 years	0.4 [0.2–0.9]	0.5 [0.2–1.0]	0.7 [0.3–1.3]		0.8 [0.4–1.7]	0.8 [0.3–1.8]	0.7 [0.4–1.2]	0.6 [0.3–1.1]	0.6 [0.3–1.5]	
**Client factors**	**Model 1c**		**Model 2c**		**Model 3c**		**Model 4c**		**Model 5c**	
**Age**										
< 20 years	1.0	1.0	1.0		1.0	1.0	1.0	1.0	1.0	1.0
20–35 years	2.3 [1.1–5.1]	**2.7 [1.2–6.0]**	1.4 [0.8–2.3]		2.1 [1.2–3.9]	**2.1 [1.1–4.1]**	1.9 [1.2–3.3]	**2.0 [1.2–3.4]**	3.3 [1.5–7.4]	**3.0 [1.3–6.9]**
> 35 years	1.6 [0.5–4.9]	2.0 [0.6–6.4]	1.1 [0.5–2.6}		1.1 [0.4–2.9]	1.0 [0.4–2.7]	1.2 [0.6–2.4]	1.2 [0.6–2.7]	5.5 [1.5–19.7]	3.6 [0.8–16.1]
**Education level**										
None	1.0	1.0	1.0		1.0	1.0	1.0	1.0	1.0	1.0
Primary	1.9 [0.9–4.1]	1.8 [0.8–4.0]	0.9 [0.5–1.7]		0.7 [0.4–1.5]	2.1 [1.1–4.1]	1.4 [0.9–2.3]	1.3 [0.8–2.2]	2.8 [1.1–7.0]	2.5 [0.9–6.7]
secondary and above	2.2 [0.8–5.8]	1.8 [0.6–5.4]	1.2 [0.6–2.4]		0.7 [0.3–1.5]	1.0 [0.4–2.7]	2.1 [1.2–3.9]	1.6 [0.9–3.0]	2.3 [0.8–6.6]	**3.0 [1.0–9.3]**

Note: All ORs in models 1d, 2d, 3d, 4d, and 5d were adjusted ORs. Each variable in these models had been adjusted by all other variables within that model.

## Discussion

The results presented here indicated an unsatisfactory level of provider adherence to first-visit ANC standards in Tanzania. In addition, at the dispensary level, the type of cadre and client age, at health centre; quality assurance, at hospital; availability of routine tests and basic medicines were significant determinants of provider adherence to first-visit ANC standards. Furthermore, at public facilities; the presence of staff who received ANC refresher training, and at private facilities; receiving external funding from the government and clients’ education level also were associated with provider adherence to first-visit ANC standards.

This unsatisfactory level of provider adherence to first-visit ANC standards in Tanzania was consistent with earlier studies conducted in Zambia and Nigeria [43,44]. The similarity of these findings may have been because both the present and previous studies analyzed nationally representative data that were collected and managed by the DHS program, so these studies had similar designs and methodologies. In this stratified analysis, when the adherence to first-visit ANC was compared across type of facility, the findings indicated that providers at higher level facilities (hospitals or health centers) showed greater adherence to ANC standards than those in lower level facilities (dispensaries or clinics). This was similar to the findings of previous studies conducted in Nigeria [44], Nepal [45] and Ghana [46–48]. The greater adherence in higher level facilities may be because these facilities are well equipped with large numbers of health workers and the ready availability of basic medicines [49–51]. However, adherence was consistent across the type of managing authority.

Despite the inadequate overall level of adherence to first-visit ANC, there was a substantial difference in performance across individual domains. In contrast to a previous study in Tanzania, the results of the present study indicated a high level of adherence by providers in the domains of client history taking and HIV testing and counselling [52].

The availability of basic routine diagnostic tests is important for providing high-quality ANC services [53]. Although more than half of the consultation providers adhered to the aspect of performing or referring pregnant women for routine diagnostic tests, few facilities reported having tests for haemoglobin (Hb) and urinary protein. This discrepancy may have been because some providers discuss the importance of having routine tests with the client although such tests were not available within the facility. Therefore, the provider may request that the client undergo the test at another health facility. This finding was consistent with those of previous studies conducted in other SSA countries indicating the inadequate availability of recommended routine diagnostic tests, including those for Hb level and urinary protein, during ANC visits [43–45,47,54,55].

A physical examination and the provision of supplements to pregnant women are recommended for pregnant women in LMIC [56]. The present study indicated relatively high levels of provider adherence in the domains of physical examination and the provision of supplements to pregnant women during ANC visits. This can be explained as the physical examinations required during the first visit involve simple and relatively inexpensive equipment, such as a tape measure to determine fundal height, manual abdominal palpations, and a fetal-scope to determine fetal heart rate in cases where gestational age is 16 weeks or longer. These findings were consistent with those of previous studies performed in Uganda [57], Nigeria [58], and India [59].

Assessment of danger signs and education of women regarding obstetric complications are important components of ANC [60,61]. Both the previous and new WHO ANC models recommend that counseling and assessment of danger signs should be conducted during each ANC visit [20]. However, previous studies performed in Tanzania indicated that the majority of pregnant women were unaware of danger signs related to pregnancy despite having attended ANC visits [62–64]. In the present study, less than half of the observed ANC consultations assessed danger signs and counseled women on danger signs. Similar findings were reported in another study performed in Tanzania [65]. The poor adherence of providers in this important domain may result in a low level of awareness among women regarding obstetric danger signs, and therefore expose them to high risks of maternal complications [57].

In the present study, we analyzed a range of factors postulated to be associated with adherence to first-visit ANC standards using separate stratified regression models according to facility type and managing authority. The results obtained from these models indicated that good adherence to first-visit ANC was strongly associated with providers at health centers that regularly performed quality assurance at least on an annual basis. This may have been because quality assurance was accompanied by continuous monitoring and persistent feedback to improve services, thus guaranteeing and maintaining a high standard of service provided by the healthcare system [66,67]. Hence emphasize should be made to promote the regular performance of quality assurance in order to improve the provider adherence to first-visit ANC standards. In addition, providers at hospitals and public facilities that had access to basic medicine and hospitals that had performed routine tests showed stronger associations with adherence to first-visit ANC standards. This was consistent with the necessity of basic inputs, such as supportive equipment and appropriate medicines, to provide high-quality services [68]. In addition, providers at private facilities that received some extra incentives from the government were strongly associated with adherence to ANC standards. Most of these facilities are operated under the Public-Private Partnerships (PPP) for Health. Through this framework, private facilities operate as public facilities and receive financial incentives and other supplies from the government to provide ANC services without charge. Therefore, providers within these facilities are more likely to adhere to ANC standards than those in completely private facilities that are operated on a for-profit basis [69].

In contrast to evidence suggesting that ANC can be managed effectively by providers other than clinicians in low-risk pregnancies [70], the present study highlighted that clinicians at lower level facilities performed better with regard to adherence to first-visit ANC standards than other providers. This may have been because the shortage of sufficient personnel at lower level facilities in Tanzania resulted into some preventive services, such as ANC, being provided by nurse auxiliaries (attendants) [71]. Despite this, nurse auxiliaries receive only minimal pre-service training of 1 year and they are also excluded from refresher training due to the MoHCDGEC plan to phase out these personnel. Therefore, they have been reported as showing the least adequate adherence to ANC guidelines [71,72]. Efforts should be made to provide more competent health professionals through competency-based approach training to overcome the health workforce shortage in low resource settings, such as Tanzania [73,74].

Refresher training for health care providers creates the opportunity to receive the up-to-date information and skills required to provide quality health care [75,76]. The results of the present study support the suggestion that refresher training guarantees better ANC care [77,78]. This may be because refresher training improved the health care providers’ knowledge and level of confidence, as these are important components in the provision of quality health services [79,80]. Such personnel may have been more likely to adhere to ANC standards of care than those who had not received refresher training or had received such training more than the 2 years previously. In contrast to our findings, a previous study using pooled data from eight countries, including Tanzania, suggested that refresher training for providers did not significantly improve the quality of ANC in that region [81].

Several studies have highlighted the impact of client characteristics on the adherence of providers to standards of care [48,82,83]. The results of the present study indicated that across almost all strata, adherence to first-visit ANC standards was better when providers attended women between 20 and 35 years old. This was similar to the findings of a previous study performed in Ghana [84]. Furthermore, our findings were consistent with those of previous studies indicating that older age was associated with higher quality ANC services [29,45], although our findings were not statistically significant.

The observed similarities between the findings of the current study and those reported in previous researches were due to the similarities in study design (both used cross-sectional survey) such as studies conducted Uganda, Nigeria, and India [57–59]. Also, the observed difference between the findings of the current study and those from previous studies conducted in Tanzania may have been due to differences in sample size and number of regions included, i.e., the present study included all 30 regions of Tanzania and large sample, while the previous study included only one region [52]. In addition, the other study used some of the data (e.g., in TSPA 2006) which were collected more than 10 years ago [81]. This time difference may explain the changes in some of the aspects such as refresher training initiatives that prioritize competencies rather than the acquisition of knowledge through short-term lectures and seminars [73,74].

If all these factors identified in this study will be addressed properly, it will provide a good start for Tanzania and other countries with similar settings to adopt and implement the new WHO 2016 ANC model in order to improve quality of ANC.

The strength of the present study was that it used the TSPA 2014–2015 dataset, which is a comprehensive national dataset, suggesting that our findings accurately reflect the current situation regarding the quality of first-visits ANC in the study setting. In addition, the present study took into account multi-level factors (facility, provider, and client) and applied stratified analysis to identify targets for policy interventions.

Nevertheless, this study also had some limitations due to its cross-sectional nature, meaning that causality could not be inferred and the results should, therefore, be interpreted with caution. Based on our selection criteria, in the majority of health facilities, only one first-visit ANC was observed. This may have compromised the representativeness of all other possible first-visits ANC at these facilities. The use of stepwise elimination methods based on the highest *P*-value to fit the final models of logistic regression analysis may have undermined the validity of the final inferences. The use of an arbitrary approach to calculate the adherence score and the cut-off point set ≥ 60.5% to dichotomize the outcome variable may have resulted in misclassification of provider adherence status. Finally, although the direct observation technique used in this study is regarded as the gold standard for assessing the quality of care, this technique is susceptible to observation biases as well as the Hawthorne effect, i.e., the alteration of behavior by the subjects of a study due to their awareness of being observed [85].

## Conclusions

The results of the present study highlighted important factors, such as refresher training, regular distribution of basic medicines, the performance of quality assurance, and diagnostics equipment, influencing provider adherence to first-visit ANC standards. The new WHO 2016 ANC model emphasizes increasing the number of ANC visits to provide respectful, individualized, and personal care at each visit. The goals of this model could be reached in Tanzania by implementation together with efforts to improve the availability of ANC services, especially in lower-level and publicly owned facilities.

## Supporting information

S1 Table A summary for the measurement of outcome variable. (DOCX)

S2 Table A summary for the measurement of independent variables. (DOCX)
