# Supplementary material for: Adherence to standards of first-visit antenatal care among providers: A stratified analysis of Tanzanian facility-based survey for improving quality of antenatal care
Source: PLoS One. 2019 May 13;14(5):e0216520. doi: 10.1371/journal.pone.0216520 (PMC6513091; doi:10.1371/journal.pone.0216520)
Supplement: S1 Table — (DOCX) [file pone.0216520.s001.docx]

**S1 Table. A summary for the measurement of outcome variable**

| **Domain and its items** | **Measurement** | **Percent score** | |
| --- | --- | --- | --- |
|  |  | Item | Domain |
| **Client history** |  |  |  |
| Client’s age | 1=Asked, 0=Not | 2.50 | 10.00 |
| Medications the client is taking | 1=Asked, 0=Not | 2.50 |  |
| Date client’s last menstrual period began | 1=Asked, 0=Not | 2.50 |  |
| Number of prior pregnancies client has had | 1=Asked, 0=Not | 2.50 |  |
| **Aspects of prior pregnancies** |  |  |  |
| Prior stillbirth(s) | 1=Asked, 0=Not | 1.00 | 10.00 |
| Infant(s) who died in the first week of life | 1=Asked, 0=Not | 1.00 |  |
| Heavy bleeding, during or after delivery | 1=Asked, 0=Not | 1.00 |  |
| Previous assisted delivery (caesarean section/vacuum/forceps) | 1=Asked, 0=Not | 1.00 |  |
| Previous spontaneous abortions | 1=Asked, 0=Not | 1.00 |  |
| Previous multiple pregnancies | 1=Asked, 0=Not | 1.00 |  |
| Previous prolonged labor | 1=Asked, 0=Not | 1.00 |  |
| Previous pregnancy-induced hypertension | 1=Asked, 0=Not | 1.00 |  |
| Previous pregnancy related convulsions | 1=Asked, 0=Not | 1.00 |  |
| High fever or infection during prior pregnancy/pregnancies | 1=Asked, 0=Not | 1.00 |  |
| **Danger signs of the current pregnancy** |  |  |  |
| Vaginal bleeding | 1=Asked, 0=Not | 1.43 | 10.00 |
| Fever | 1=Asked, 0=Not | 1.43 |  |
| Headache or blurred vision | 1=Asked, 0=Not | 1.43 |  |
| Swollen face or hands or extremities | 1=Askedd, 0=Not | 1.43 |  |
| Tiredness or breathlessness | 1=Asked, 0=Not | 1.43 |  |
| Cough or difficulty breathing for 3 weeks or longer | 1=Asked, 0=Not | 1.43 |  |
| Any other symptoms or problems related to this pregnancy | 1=Asked, 0=Not | 1.43 |  |
| **Physical examination** |  |  |  |
| Take the client’s blood pressure | 1=Performed, 0=Not | 1.25 | 10.00 |
| Weigh the client | 1=Performed, 0=Not | 1.25 |  |
| Examine conjunctiva/palms for anemia | 1=Performed, 0=Not | 1.25 |  |
| Examine legs/feet/hands for edema | 1=Performed, 0=Not | 1.25 |  |
| Examine for swollen glands or lymphnodes | 1=Performed, 0=Not | 1.25 |  |
| Palpate or measure the client’s abdomen for fundal height | 1=Performed, 0=Not | 1.25 |  |
| Examine the client's breasts | 1=Performed, 0=Not | 1.25 |  |
| Conduct vaginal examination/exam of perineal area | 1=Performed, 0=Not | 1.25 |  |
| **Routine tests** |  |  |  |
| Hemoglobin test | 1=Performed, 0=Not | 2.50 | 10.00 |
| Blood grouping | 1=Performed, 0=Not | 2.50 |  |
| Any urine test | 1=Performed, 0=Not | 2.50 |  |
| Syphilis test | 1=Performed, 0=Not | 2.50 |  |
| **HIV Testing and Counseling** |  |  |  |
| Asked if the client knew her HIV status | 1=Asked, 0=Not | 2.00 | 10.00 |
| Provide or refer for counseling related to HIV test | 1=Performed, 0=Not | 2.00 |  |
| Perform or refer for HIV test | 1=Performed, 0=Not | 2.00 |  |
| Provided post test counseling | 1=Performed, 0=Not | 2.00 |  |
| Discussed about partner testing | 1=Discussed, 0=Not | 2.00 |  |
| **Maintain health pregnancy** |  |  |  |
| Discussed nutrition (i.e., food to eat) during the pregnancy | 1=Discussed, 0=Not | 3.33 | 10.00 |
| Informed the client about the progress of the pregnancy | 1=Performed, 0=Not | 3.33 |  |
| Discussed the importance of at least 4 ANC visits | 1=Performed, 0=Not | 3.33 |  |
| **Iron/ Folate (FeFo) supplementation** |  |  |  |
| Prescribed or gave iron pills or folic acid (FeFo) or both | 1=Performed, 0=Not | 2.50 | 10.00 |
| Explained the purpose of iron or folic acid (FeFo) | 1=Performed, 0=Not | 2.50 |  |
| Explained how to take iron or folic-acid (FeFo) pills | 1=Performed, 0=Not | 2.50 |  |
| Explained side effects of iron or folic-acid (FeFo) pills | 1=Performed, 0=Not | 2.50 |  |
| **Tetanus toxoid injection** |  |  |  |
| Prescribed or gave a tetanus toxoid (TT) injection | 1=Performed, 0=Not | 3.33 | 10.00 |
| Explained the purpose of the TT injection | 1=Performed, 0=Not | 3.33 |  |
| Checked TT card/ANC card | 1=Performed, 0=Not | 3.33 |  |
| **Preparation for delivery** |  |  |  |
| Asked the client where she will deliver | 1=Asked, 0=Not | 2.00 | 10.00 |
| Advised the client to prepare for delivery | 1=Advised, 0=Not | 2.00 |  |
| Advised the client to use a skilled health worker for delivery | 1=Advised, 0=Not | 2.00 |  |
| Advise what items to have in hands in case of emergency | 1=Advised, 0=Not | 2.00 |  |
| Advised the client to deliver at a health facility | 1=Advised, 0=Not | 2.00 |  |
| **Total ANC adherence score** |  |  | **100.00** |
