# Supplementary material for: Adherence to standards of first-visit antenatal care among providers: A stratified analysis of Tanzanian facility-based survey for improving quality of antenatal care
Source: PLoS One. 2019 May 13;14(5):e0216520. doi: 10.1371/journal.pone.0216520 (PMC6513091; doi:10.1371/journal.pone.0216520)
Supplement: S2 Table — (DOCX) [file pone.0216520.s002.docx]

**S2 Table. A summary for the measurement of independent variables**

| **Variable** | **Measurement** |
| --- | --- |
|  |  |
| **Facility location** | 1 = “Urban;” for the facilities that were located in urban area |
|  | 0 = “Rural;” for the facilities that were located in urban area |
| **Facility type** | 1 = “Clinic/ Dispensary;” for facilities that were either clinic or dispensary |
|  | 2 = “Health centre;” if the facility was health centre |
|  | 3 = “Hospital;” if the facility was hospital |
| **Managing authority** | 1 = “Public;” for the facilities that were owned by government |
|  | 0 = “Private;” for the facilities that were owned by private institutions, mission, faith-based, and other institution rather than government |
| **Quality assurance** | 1 = “Performed;” for facility that reported routinely carry out quality assurance activities e.g., review of mortality, or audit of registers within 12 months. |
|  | 0 = “Not performed;” for facility that reported not routinely carry out quality assurance activities within 12 months |
| **ANC guideline** | 1 = “Available;” for facilities observed having ANC national guideline or other ANC guidelines |
|  | 0 = “Not available;” for facilities observed not having ANC national guideline or other ANC guidelines |
| **Basic equipment** | 1 = “Available;” for facilities observed having either digital BP apparatus or manual BP apparatus with stethoscope |
|  | 0 = “Not available;” for facilities observed tot having either digital BP apparatus or manual BP apparatus with stethoscope |
| **Basic medicines** | 1 = “Available;” for facilities observed having Iron and Folate supplements, Sulfadoxine + Pyrimethamine(SP), Tetanus toxoid vaccine, and Insecticide Treated Net (ITNs) |
|  | 0 = “Not available;” for facilities observed not having either Iron and Folate supplements, Sulfadoxine + Pyrimethamine(SP), Tetanus toxoid vaccine, or Insecticide Treated Net (ITNs) |
| **External source of fund** | 1 = “Government;” for facilities that reported to receive some fund from government despite the type of managing authority |
|  | 0 = “Non-government;” for facilities that not receiving any fund from government |
| **Provider’s sex** | 1 = “Female;” if provider was female |
|  | 0 = “Male;” if provider was male |
| **Cadre** | 1 = “Nurse;” if the provider was registered nurse, enrolled nurse, nurse assistant, or attendant |
|  | 0 = “Clinician;” if the provider was general medical doctor, specialist medical doctor, assistant medical officer, clinical officer, or clinical assistant |
| **Refresher training** | 1 = “Received;” if the provider have received any in-service training, training updates or refresher training about ANC within the past 24 months |
|  | 0 = “Not received;” if the provider have not received such kind of trainings |
| **Supportive supervision** | 1 = “Not received;” if the provider have not received supportive supervision |
|  | 2 = “Received <3 months;” if the provider have received supportive supervision from seniors within 3 months from the day of interview |
|  | 3 = “Received >3 months;” if provider received supportive supervision 3 months or more from the day of interview |
| **Working experience** | 1 = “< 2 years”; if the providers has less than two year working experience |
|  | 2 = “2 – 5 years;” if the provider has working experience of two up to five years |
|  | 3 = “>5 years;” if the provider has more than five years working experience |
| **Age** | 1 = “< 20 years;” if the client had age of less than 20 years |
|  | 2 = “20 – 35 years” if the client had age between 20 and 35 years |
|  | 3 = “> 35 years” if the client had age more than 35 years |
| **Education level** | 1 = “None” if the client has not attended any kind of formal education |
|  | 2 = “Primary” if the client has attended primary level of education |
|  | 3 = “secondary and above” if the client has attended either secondary, vocational training, college, or university |
